# Sensory sensitivity as a link between concussive traumatic brain injury and PTSD

**DOI:** 10.1038/s41598-019-50312-y

**Published:** 2019-09-25

**Authors:** Ann N. Hoffman, Jamie Lam, David A. Hovda, Christopher C. Giza, Michael S. Fanselow

**Affiliations:** 1 UCLA, Neurosurgery; Brain Injury Research Center, Los Angeles, USA; 2 UCLA, Psychology, Los Angeles, USA; 3 UCLA Steve Tisch BrainSPORT Program, Los Angeles, USA; 4 UCLA, Medical and Molecular Pharmacology, Los Angeles, USA; 5 UCLA Mattel Children’s Hospital, Los Angeles, USA; 6 UCLA, Psychiatry and Biobehavioral Sciences, Los Angeles, USA; 7 Staglin Center for Brain and Behavioral Health, Life Sciences, UCLA, Los Angeles, USA

**Keywords:** Brain injuries, Post-traumatic stress disorder

## Abstract

Traumatic brain injury (TBI) is one of the most common injuries to military personnel, a population often exposed to stressful stimuli and emotional trauma. Changes in sensory processing after TBI might contribute to TBI-post traumatic stress disorder (PTSD) comorbidity. Combining an animal model of TBI with an animal model of emotional trauma, we reveal an interaction between auditory sensitivity after TBI and fear conditioning where 75 dB white noise alone evokes a phonophobia-like phenotype and when paired with footshocks, fear is robustly enhanced. TBI reduced neuronal activity in the hippocampus but increased activity in the ipsilateral lateral amygdala (LA) when exposed to white noise. The white noise effect in LA was driven by increased activity in neurons projecting from ipsilateral auditory thalamus (medial geniculate nucleus). These data suggest that altered sensory processing within subcortical sensory-emotional circuitry after TBI results in neutral stimuli adopting aversive properties with a corresponding impact on facilitating trauma memories and may contribute to TBI-PTSD comorbidity.

## Introduction

Traumatic brain injury (TBI) is one of the most common injuries to military personnel, a population often exposed to stressful stimuli and emotional trauma. TBI typically impairs learning and memory for neutral events^1^, but may enhance traumatic fear memories^2^. Furthermore, long-term consequences of TBI include increased risk for neurological and psychiatric disorders^3–7^. Increasing numbers of military service members that have been exposed to both emotional and physical trauma have promoted interest in understanding the comorbidity between TBI and post-traumatic stress disorder (PTSD), as mild TBI is a significant predictor of PTSD following deployment^8^. It remains a clinical challenge to know which precipitating event causes PTSD in TBI, the brain injury itself or the psychological stressor(s) surrounding the TBI. Understanding how and whether traumatic memories are encoded differently after TBI will aid in our efforts in recognizing underlying mechanisms and developing effective treatments for this highly prevalent and complex comorbidity. Here we combined widely used preclinical models to identify potential causal links between TBI and PTSD.

Pavlovian fear conditioning is widely used to study fear learning and memory, where an innocuous stimulus (conditional stimulus, CS), such as an auditory cue is paired with an aversive stimulus (unconditional stimulus, US), such as a footshock, which promotes natural defensive responses. Following association between CS and US, both the context and CS alone will elicit a learned conditional response (CR), freezing, indicative of fear. The basolateral amygdala complex is a key locus of plasticity for the formation of fear memories^9^. The lateral subdivision (lateral amygdala, LA), which receives direct cortical and thalamic auditory input, is known as the auditory interface of the amygdala and is required for auditory fear conditioning^10^. While the amygdala has been relatively understudied in TBI research, recent data from our lab and others have begun to find TBI-induced changes in amygdala structure and function towards enhanced excitatory processes and increased plasticity^11–14^. Specifically, we reported that 48 h after fluid percussion injury (FPI), an experimental model of diffuse concussive-like TBI, rats displayed enhanced contextual fear learning when footshocks were signaled with a white noise CS, but not when unsignaled^11^. These enhanced fear memories were associated with increased N-methyl-D-aspartate (NMDA) receptor expression in the BLA^11^. The hippocampus also plays a key role in the formation of contextual fear memories^15–17^, has significant implications in PTSD^18–20^, and is highly susceptible to dysfunction following TBI^21–23^. Thus the neural circuits necessary for encoding adaptive auditory fear memories are vulnerable to disruption after TBI and may lead to increases in fear learning and expression^24^.

Sensory sensitivity or sensory processing issues including photophobia (light sensitivity), phonophobia (sound sensitivity), hyperacusis (sensitivity to certain sound frequencies), and allodynia (tactile sensitivity) are among the primary physical symptoms of diffuse clinical TBI or concussion^25–27^, and are often categorized and treated separately from neuropsychiatric sequelae^28^. Experimental evidence suggests that somatosensory input is enhanced after injury^29,30^. Interactions between aberrant sensory processing and amygdala vulnerability in the TBI brain may impact stress reactions and underlie potential mechanisms of comorbid TBI/PTSD. By separating the physical from emotional trauma, our design provides the opportunity to determine whether effects such as sensory sensitivity from the TBI itself impact function in networks required for fear memory encoding. We hypothesized that FPI enhances contextual fear to auditory fear conditioning as a result of injury-induced altered auditory processing. In this study, we examined the stimulus-specific enhancement of contextual fear learning after FPI and changes in projection-specific functional plasticity in auditory fear circuitry and the hippocampus.

## Results

### After FPI, white noise, but not pure tone, evokes robust defensive behavior and enhances contextual fear when used in fear conditioning

**Figure 1:**
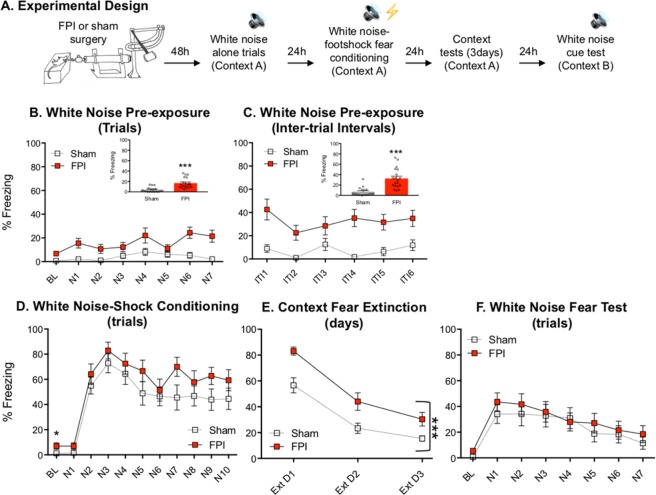
White noise promotes defensive behavior after fluid percussion injury and contributes to enhanced contextual fear learning when paired with footshocks. (**A**) Experimental design. (**B**) During noise pre-exposure with presentations of white noise cues in the absence of footshocks, FPI (fluid percussion injury) groups froze significantly more than shams and (**C**) to an even greater degree at the offset and during the intervals between trials. (**D**) When white noise was paired with footshocks, both groups increased freezing across trials indicating learning, however the FPI group was not different than sham. (**E**) Freezing in the conditioning context was robustly increased in the FPI groups across three days of testing. Both groups decreased freezing across days, indicating contextual fear extinction. (**F**) When tested in a novel context there were no group differences in freezing to the white noise cue. ***p < 0.001 vs. Sham; data are represented as mean ± SEM.

**Figure 2:**
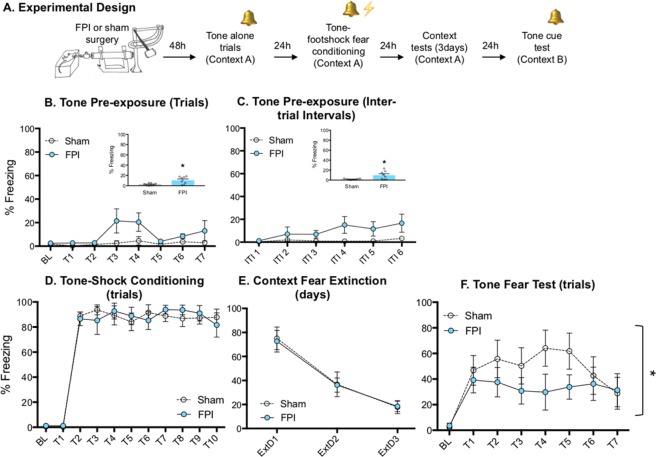
Lateral fluid percussion injury reduces tone fear memory. (**A**) Experimental design. (**B**,**C**) FPI (fluid percussion injury) rats displayed slightly elevated levels of freezing behavior during pre-exposure to pure tone (2800 Hz/75 dB) trials **(B)** and during inter stimuli intervals (**C**). (**D**) FPI had no effect on baseline freezing prior to the first tone-shock conditioning trial. Although both groups learned, FPI had no effect on freezing across acquisition trials when pure tones were paired with mild footshocks. (**E**) While both groups decreased freezing across context extinction sessions, FPI had no effect on fear to the conditioning context. (**F**) FPI rats froze less during tone CS trials when presented in a novel context. *p < 0.05 vs. Sham. Data are represented as mean ± SEM.

We sought to determine the effects of FPI on auditory and contextual fear memory with white noise used as a CS paired with shock. Outlined in Fig. 1A, two days following either FPI or sham surgery, rats were pre-exposed to white noise CS cues (30 sec/75 dB) and the following day received 10 white noise-shock pairings. Two cohorts of animals were used and data were combined in this experiment for replicability (sham, n = 16; FPI, n = 19). When white noise was presented alone, FPI groups showed increased freezing during cue presentations compared to shams (F(1, 33) = 21.055, p < 0.001; Fig. 1B), and this response was most robust during the post-noise intervals between trials (F(1, 33) = 24.417, p < 0.001; Fig. 1C). The next day, both groups were fear conditioned with 10 white noise-footshock pairings in the same context. Although at low levels (sham, 1.4 ± 2.1%; FPI 6.9 ± 10.4%), FPI groups displayed significantly more freezing during the baseline period prior to the first CS-US trial compared to sham (t(29) = 2.081, p = 0.046; BL: Fig. 1D)., indicating pre-existing unconditional responding from white noise influenced context freezing prior to paired presentations with footshocks. Successful acquisition was indicated by increased freezing over conditioning trials (F(9, 216) = 22.265, p < 0.001; Fig. 1D), however group differences during conditioning did not reach statistical significance (F(1, 29) = 2.513, p = 0.124), Fig. 2D. Over the next three days, both groups were tested for contextual fear extinction in the absence of discrete stimuli, both groups decreased freezing to context across days of testing (F(2, 66) = 113.535, p < 0.001). However, FPI groups exhibited robustly enhanced fear to the training context compared to shams (F(1, 33) = 12.637, p = 0.001), Fig. 1E, consistent with our previous finding^11^. When tested for white noise CS fear in a new context after levels of fear were decreased by context extinction, there was no effect of injury (F(1, 33) = 0.290, p = 0.594), however there was a significant decrease in freezing across the cue trials (F(6, 198) = 12.298, p < 0.001, Fig. 1F).

Phonophobia and hyperacusis are common and often persistent physical symptoms after TBI or concussion^28,31,32^. Therefore, we hypothesized that injury-induced altered sensory processing led to the phonophobia-like phenotype and enhanced contextual fear also found by Reger *et al*.^11^. Because white noise is comprised of a mixture of all frequencies it may differentially elicit phonophobic and/or hyperacoustic reactions. To test for stimulus specificity of the injury effect, we tested a new cohort of FPI and sham rats under the same protocol using low frequency pure tones at the same intensity (30 sec/2800 Hz/75 dB). During pre-exposure, FPI rats froze some during tone trials (F(1, 14) = 8.693, p = 0.011; Fig. 2B), and between trials (F(1, 14) = 7.767, p = 0.015; Fig. 2C) compared to sham, with an overall significant increase across trials (F(6, 84) = 2.242, p = 0.047). However, the magnitude of the effect was far greater to white noise, and in particular following the offset of the stimulus during the post-noise interval. When analyzed together by a two way mixed factors ANOVA, we saw a significant injury x stimulus interaction for pre-exposure inter-trial-intervals (ITI; F(1, 47) = 4.666, p = 0.036); FPI-tone vs. FPI-white noise post hoc comparison (F(1, 23) = 4.272, p = 0.05), Supplemental Fig. S2), indicating a greater photophobic-like response to white noise above tones. When tested the next day, baseline freezing did not differ between injury conditions after pre-exposure to pure tones as it did in the first experiment following white noise (t(14) = 1.183, p = 0.256; BL: Fig. 2D). During conditioning, both groups increased freezing across acquisition trials throughout the session (F9, 126) = 50.22, p < 0.001), however both groups reached ceiling after the first trial, Fig. 2D. Over the next three days, both groups were returned to the same context to measure contextual fear and extinction to the conditioning context. While both groups decreased freezing across context extinction days (F(2, 42) = 26.9, p < 0.001), there was no effect of injury on freezing to the context (Fig. 2E). When both groups were placed in a novel context and presented with the trained tone CS, FPI rats displayed significantly less freezing during the tone test (F(1, 98) = 5.453, p = 0.0216; Fig. 2F), indicating a deficit in recall of the trained cue, which is in contrast to our previous finding^11^. These data provide novel evidence that after diffuse TBI, white noise alone evokes freezing defensive behavior. This in turn leads to enhanced contextual fear when used as a CS, consistent with our initial findings with white noise^11^, while tone fear memory may be impaired.

### White noise exposure causes increased amygdala, but reduced hippocampal activity following FPI

**Figure 3:**
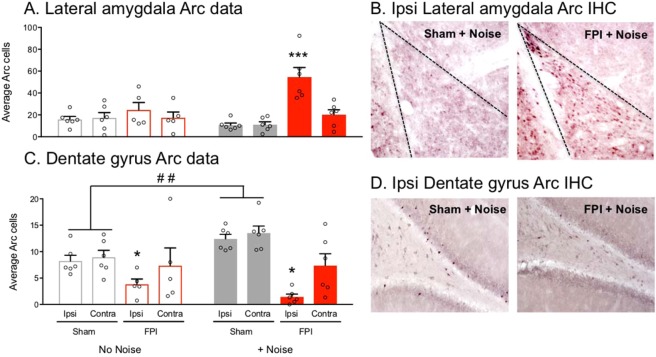
Arc protein induction in response to white noise after FPI. (**A**) Lateral amygdala, (LA) White noise exposure in FPI (fluid percussion injury) rats caused robust Arc induction within the ipsilateral LA relative to contralateral and all other groups, ***p < 0.001 vs. contra and all other groups. (**C**) Dorsal dentate gyrus, (DG) FPI caused a significant overall reduction in Arc protein in response to novel context exploration alone or in the presence of white noise exposure in the ipsilateral DG (*p < 0.05). White noise exposure led to greater Arc induction within the dorsal DG in uninjured sham controls (^##^p < 0.01), but not FPI groups. Data are represented as mean ± SEM. (**B**,**D**) Photomicrographs are representative images of Arc immunohistochemistry (IHC) within the ipsilateral LA (**B**) and DG (**D**) in groups exposed to white noise.

Our behavioral data suggest that FPI makes white noise in and of itself aversive, or “US-like.” We then asked how the injured brain responds differently to white noise using activity dependent Arc immunolabeling within important targets including the lateral amygdala (LA) and dorsal dentate gyrus (DG) of the hippocampus. The LA receives direct sensory information from auditory cortical and thalamic areas, which is required for the formation of auditory fear^10^. The dorsal DG is highly vulnerable to insult after TBI^33^ and required for contextual fear memory formation^34^. Two days following FPI or sham surgery, animals were placed in a novel context and exposed to either 7 white noise alone trials, similar to the pre-exposure sessions in earlier experiments (75 dB), or were quiet controls and brains were processed for Arc immunohistochemistry IHC (n = 5–6/group). Arc is rapidly expressed in projection neurons as a result of a single behavioral experience^35^, and is necessary for associative fear memories in the amygdala^36,37^. Interestingly, two way between subjects multivariate ANOVA revealed a significant injury effect (Wilks’ Lambda (F(4, 16) = 21.14, p < 0.001), and an injury x noise condition multivariate interaction (Wilks’ Lambda (F(4, 16) = 5.242, p = 0.007) for Arc positive cells across ipsi- and contralateral lateral amygdala (LA) and dorsal dentate gyrus (DG). Between subjects MANOVA effects showed a significant injury x noise condition effect within the ipsilateral LA (F(1, 19) = 13.465, p = 0.002), where posthoc analyses showed that only noise-exposed FPI rats had robust increased Arc expression relative to all other groups, (p < 0.05; Fig. 3A). Between subjects MANOVA revealed that in the DG of the hippocampus there was also a significant injury x noise condition interaction. Post hoc analyses for sham controls showed that regardless of hemisphere, there was a significant effect of noise condition (Wilks’ Lambda, F(4, 7) = 13.052, p = 0.002), where noise-exposed shams had more Arc induction than quiet controls, Fig. 3C. This was not the case in FPI animals, where regardless of noise condition, there was significantly less Arc induction in the ipsilateral hemisphere (F(1, 19) = 73.961, p < 0.001), which is in direct contrast to effects observed in the LA, Fig. 3C. This pattern of findings suggests dysregulated activity within the limbic regions that are important for processing contextual fear in the presence of white noise stimuli that in the absence of FPI are not aversive or noxious.

### Increased activity in ipsilateral thalamo-amygdala projecting neurons during white noise exposure after lateral FPI

**Figure 4:**
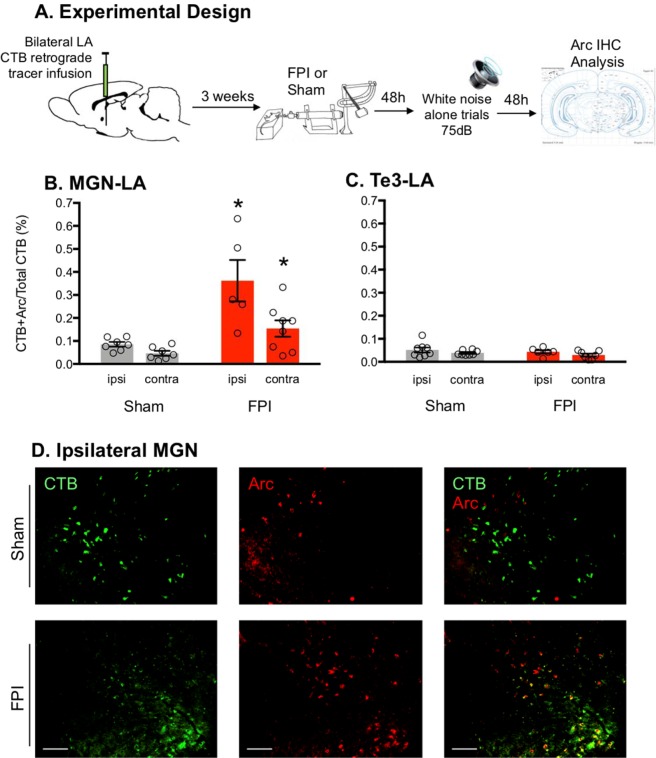
Increased activity in ipsilateral thalamo-amygdala projecting neurons during white noise exposure after lateral FPI. (**A**) Experimental design. (**B**) Increased Arc activity in MGN-LA (medial geniculate nucleus-lateral amygdala) projection neurons (*p < 0.05), but not Te3-LA (secondary auditory cortex-lateral amygdala; **C**) during white noise exposure after FPI. *p < 0.05; Data are represented as mean ± SEM. (**B**) Representative Arc activity (red) in retrolabeled CTB (cholera toxin subunit B) in LA afferents (green) in ipsilateral MGN. Scale bar 200 µm.

The LA receives direct sensory input from cortical and thalamic regions that drive plasticity and are known to be required for auditory fear learning^38^. In order to determine which amygdala inputs may be disrupted following FPI that drive the increased activity in the LA, we measured Arc expression in MGN-LA and Te3-LA projecting neurons during white noise exposure 48 h after lateral FPI in rats that had previously received bilateral LA retrograde tracer infusions with CTB (Fig. 4A). Replicating earlier experiments, FPI rats froze significantly greater in the context than shams between white noise trials (75 dB; t(17) = 3.711, p = 0.0017). Data included in CTB + Arc analyses were only from subjects with accurate LA CTB placement verified by expression in infusion site and by ipsilateral LA afferent retrograde CTB expression (sham ipsi, n = 9; sham contra, n = 8; FPI ipsi, n = 5; FPI contra, n = 8). MANOVA revealed a significant effect of injury for LA projecting cells in the MGN that were positive for Arc (F(2, 7) = 9.275, p = 0.011; Fig. 4B), and between group effects supported increased MGN-LA Arc activity in both ipsi (F(1, 8) = 9.509, p = 0.015) and contra (F(1, 8) = 9.175, p = 0.016) indicating that there was a significant increase in activity within MGN-LA cells in FPI compared to sham; Fig. 4B). There was no significant multivariate effect for Arc for the secondary auditory cortex (Te3)-LA (Fig. 4C). This effect was corroborated with a different IEG also involved in synaptic plasticity, c-Fos (see Supplemental Fig. S4), indicating that white noise after FPI specifically increased MGN-LA activity that may drive increased amygdala plasticity and underlie corresponding sensitivity to white noise and elicited defensive behavior.

## Discussion

Combining well established models of brain trauma and emotional trauma, this study investigated the novel role of auditory sensitivity on enhanced learned fear following diffuse TBI. Our data showed that lateral FPI robustly increased contextual fear when white noise was used as a cue paired with footshocks (US), but not with pure tone cues. We also found that in the absence of footshocks, 75 dB white noise elicited phonophobia-like defensive behavior (freezing) following FPI. Furthermore, we found increased induction of Arc protein within the ipsilateral lateral amygdala (LA) only in white noise-exposed FPI groups relative to sham and quiet FPI controls, indicating that the white noise alone initiates greater amygdala activity after brain injury. We then determined that increased activity in thalamo-amygdala projection neurons were more active during white noise on the injured hemisphere. We also observed a reduction in Arc activity within the dorsal dentate gyrus (DG) of the hippocampus in injured animals during white noise exposure, indicating dysregulated network function within brain regions required for the formation of contextual and auditory fear memories. Collectively, our data indicate that lateral FPI led to sensitivity to white noise that significantly facilitated the formation of contextual fear memories and amygdala activity during the early phase following injury.

### White noise is aversive after FPI

Contextual fear memories are most robust when the US (a footshock) is unsignaled or unpredictable^39–41^. When the shock is preceded by an auditory stimulus as a CS, the CS is predictive of the shock and therefore more strongly conditioned than the context in which the experience occurred, although there is still a degree of conditioning to the context itself. The contextual memory is known to require both the hippocampus and amygdala^42,43^. Our current and previous data support that the context is more strongly conditioned after FPI when white noise is used as a CS^11^. This white noise-specific effect is also supported by our behavioral data indicating that FPI had no effect on contextual fear during baseline after pure tone pre-exposure or following tone-shock fear conditioning. Furthermore, our previous study showed that FPI did not affect contextual fear memory when footshocks were unsignaled^11^. At higher decibels, white noise functions as a US^44–47^. Emerging evidence suggests differential plasticity and defensive behavior patterns in response to white noise and pure tones in auditory fear conditioning^48,49^, and may even recruit additional circuitry^50^. Given that white noise is a mixture of all frequencies it may differentially elicit phonophobic and/or hyperacoustic reactions following FPI. Perhaps at a lower intensity and/or within frequency ranges captured, white noise may be processed as aversive after TBI. We also observed increased Arc induction in the ipsilateral LA in the FPI group that received noise exposure. Importantly, LA neurons receive US-evoked excitatory inputs from both thalamic and cortical areas^51,52^. Auditory processing changes have also been observed in human concussion and may serve as a potential biomarker by way of evoked frequency-following response^26^. Among clinical risk factors for persistent post-concussion symptoms in a large pediatric cohort, only noise sensitivity, headache, and fatigue were included in the final predictive model^27^^,^see also^53^. Human brain imaging studies show elevated amygdala activity in response to aversive auditory stimuli^54,55^. The sensory processing difference in the FPI group may support an additive effect across cumulative exposure to both aversive white noise and footshocks that lead to overall enhanced conditional fear to the context and reflects the phonophobia-like phenotype.

### Opposing activity within emotional and cognitive neural substrates after FPI

In response to white noise, we found altered and opposing levels of functional activity within the sensory amygdala and hippocampal subregions between FPI and sham controls. We observed overall reduced Arc activity within the granule cell layer of the dorsal DG after injury. Within the sham groups, white noise resulted in increased dorsal DG Arc induction in white noise-exposed compared to quiet controls. This difference that was absent in the FPI groups perhaps reflects greater elemental learning about the context in the presence of an innocuous auditory cue to the uninjured brain, as it is understood that DG signaling is involved in contextual pattern separation^56^. Furthermore, Arc is induced in hippocampal and cortical calcium/calmoldulin-dependent protein kinase II (CaMKII) neurons during context exploration^57^ that promotes functional plasticity in memory consolidation, which is compromised after FPI. Reduced Arc observed in the injured DG is consistent with the literature regarding hippocampal function and plasticity after brain injury^58^, including reports of impairments in hippocampal LTP^59,60^, hippocampal-dependent learning and memory^61^, and reduced synaptic morphology^22^. Curiously, the FPI group exhibited *reduced* activity in the dorsal DG but also had *enhanced* context fear under similar conditions after noise-shock conditioning. While we know that the hippocampus is critical for contextual fear^62^, it is important to consider that our TBI model does not produce a lesion, but a dysfunctional hippocampal system, which in some cases leads to aberrant enhanced fear responses^63^. In contrast to the reduced activity in the injured DG, white noise exposure caused increased activity in the ipsilateral sensory amygdala (LA) in the FPI group, which may be driving the enhanced fear learning. Importantly, this effect in the LA was not observed in the quiet FPI group, supporting a stimulus-elicited effect in LA neurons that may reflect the phonophobia-like behavior in this group. Our projection mapping data suggests that this increase is driven by increased activity within ipsilateral auditory thalamus (MGN) neurons projecting to the LA. This effect was specific to the MGN and not secondary auditory cortex (Te3)-LA projections, indicating the specificity of the inputs that drive increased plasticity and corresponding defensive behavior. We also found increased astrocyte reactivity in FPI groups as measured by glial fibrillary acidic protein (GFAP) in bilateral LA, DG, and auditory cortex (Supplemental Fig. S3), and in ipsilateral MGN. Reactive glia as a result of injury may contribute to sensitized plasticity in auditory fear neural circuitry and is a target of future research. Together, our Arc data suggest an injury-induced mismatch between reduced activity in structures vulnerable to TBI that coordinate cognitive processes and context learning amid increased activity in thalamo-amygdala projections, which are known to drive fear and defensive behavior and is implicated in PTSD.

### TBI interactions with sensory-emotional network function

Auditory consequences after TBI are common in military populations, and resulting conditions like tinnitus may complicate post traumatic stress and emotional-sensory networks^64^. Our study is the first to show an interaction between sensory sensitivity and increased amygdala activity underlying defensive behavior after TBI. Consistent with our Arc findings, experimental models of tinnitus and hyperacusis have been linked to lateral amygdala hyperactivity during exposure to auditory stimuli^65^. Furthermore, sensory sensitivity has also been reported in other modalities using midline FPI, where whisker stimulation causes behavioral morbidity and elevated stress response^29^. Importantly, our findings may support an underlying mechanistic link with recent clinical studies in human TBI and other clinical populations with sensory symptoms. After TBI, anxiety interactions with sensory disturbances may underlie persistent clinical symptoms. One study assessed 12 different symptoms in physical, cognitive, and emotional categories, and found that noise sensitivity and anxiety were the only two initial TBI symptoms that were significant predictors for persistent post concussive syndrome^66^. Furthermore, another study showed that patients with chronic TBI showed persistent auditory sensitivity in a subset patients that were comorbid with PTSD^67^. Additional recent work by Papesh *et al*. showed generalized dysfunctional sensory gating and failure to habituate to auditory tones following blast exposure^68^. In other patient populations with sensory sensitivity such as in autism spectrum disorder, greater amygdala activity^55^ and thalamo-amygdala connectivity corresponds to degree of sensory overresponsitivity severity^69^. Whether interactions between sensory sensitivity and emotional reactivity and fear have a bidirectional relationship contributing to challenges in recovery after TBI remains to be determined and using animal models can allow for systematic comparison.

## Conclusions

We report that auditory sensitivity after diffuse TBI may contribute to enhanced fear learning and amygdala hyperactivity. We observed dysregulated activity between the hippocampus and thalamo-amygdala projections in response to white noise, which corresponded with a phonophobic behavioral phenotype. This led to a stimulus-specific enhancement in fear learning after white noise cued fear conditioning. These data add to the developing body of literature aimed at understanding the complexity of neurobiological interactions between comorbid TBI and PTSD^24^. Extending to other neural systems, it has also been hypothesized that changes in sensory processing after TBI may also affect cognitive and motor deficits^70^ and may contribute to increased post traumatic headache and migraine^71^. Elucidating the mechanisms that are affected in sensory and emotional systems at the circuit, cellular, and molecular levels are critical next steps in understanding how the injured brain perceives and reacts to environmental stimuli differently. In summary, our data provide implications for altered sensory processing after TBI, where otherwise neutral stimuli may adopt aversive properties and impact encoding of traumatic memories.

## Methods

### Subjects and lateral fluid percussion injury (FPI)

Young adult male Sprague-Dawley rats (Envigo, 250–275 g upon arrival) were pair housed and maintained on a 12 hour light/dark cycle with *ad libitum* food and water. All rats were handled for 1 min/day 4 days prior to surgery. All procedures were conducted with approval from the University of California Los Angeles Institutional Care and Use Committee and were in compliance with the National Institutes of Health Guide for the Care and Use of Laboratory Animals. Young adult males were utilized due to epidemiological data supporting that males are at a significantly higher risk for TBI, with the highest male-to-female ratios occurring in young adulthood^72^. Rats underwent either sham surgery or mild-moderate lateral fluid percussion injury (FPI). Lateral FPI is a general brain movement injury that exposes the entire brain to forces generated by the percussion^73–75^. FPI was induced using a previously published protocol^11,76–78^ typically used in our laboratory. Animals were anesthetized under a 1–2% isoflurane-oxygen mixture. A midline incision was made followed by a left hemisphere 3 mm diameter craniotomy centered 3 mm posterior and 6 mm lateral to bregma. A plastic injury cap was adhered to the skull with silicone gel and dental cement and filled with sterile saline. The animal was removed from anesthesia and the injury cap was attached to the fluid percussion injury device (Virginia Commonwealth University, Richmond, Virginia). Upon toe pinch response, a brief fluid pulse (~20 msec) of saline was administered directly to the dura mater. Apnea and loss of consciousness (LOC; measured by toe pinch response) were measured to determine injury severity (Fig. S1). Rats were then placed back on anesthesia to remove injury cap and suture the scalp. Sham animals received the same surgical procedures except fluid pulse impact. Upon completion of surgery, animals were placed in a heated recovery chamber until normal behavior resumed and returned to the vivarium. Animals were weighed and monitored post operatively for at least a week after surgery or until the end of the experiment. Injury severity as measured by toe pinch withdrawal was within the mild-moderate range, similar across experiments (269.2 sec mean ± 142.33 sec standard deviation) and counterbalanced within experiments comparing noise and no noise exposure after FPI (Supplemental Fig. S1).

### Fear conditioning and behavior

All behavioral testing began 48 h after surgery based on our initial findings on amygdala function following FPI^11,74^. Training and auditory cue testing occurred in two distinct conditioning chambers (context A and context B) that differed in transport, location, odor, lighting, chamber shape, and flooring (Med Associates Inc., Georgia, VT). Percent time freezing to auditory stimuli and context were recorded as measures of auditory cued and contextual fear, respectively. Behavioral testing protocols differed slightly depending on the goals of the experiments, as outlined below.

To examine the stimulus-specificity of FPI enhanced fear, rats were pre-exposed to seven trials of either pure tones (46 sec/2800 Hz/75 dB) or white noise (46 sec/75 dB) CSs one day prior to training. Auditory fear conditioning occurred in the same context (context A) and was based on the strong delay protocol in Reger *et al*., 2012 (10 CS-US pairings/46 sec CS; 2 sec/0.9 mA footshock US), which produced increased contextual fear conditioning after FPI. Over the following three days, all groups were tested for contextual fear extinction (context A; 20 min/day). The next day, both groups were tested in context B for auditory cue fear memory with 7 CS-alone trials. Experimental designs for behavioral experiments are outlined in Figs 1A and 2A.

### Arc immunohistochemistry

To examine the effects of how white noise is processed in the injured brain within auditory fear neurocircuitry, a 2 × 2 design was used where 48 h after surgery, FPI and sham rats were presented with white noise stimuli (7 trials/46 sec/75 dB) or quiet context exposure for the same duration. Ninety minutes later, all animals were euthanized and brains were processed for immediate early gene immunohistochemistry for activity-regulated cytoskeletal-associated protein (Arc) protein. Briefly, 90 min following white noise or chamber exposure, rats were retrieved from home cages and anesthetized with isoflurane until respiration ceased and were then transcardially perfused with 0.1 M phosphate buffered saline (PBS) followed by 4% paraformaldehyde. Brains were collected and postfixed in 4% paraformaldehyde for 2 h and subsequently cryoprotected in 30% sucrose over 2–3 d then flash frozen and stored in −80 °C until sectioning. Frozen sections (40 μm) containing the amygdala and dorsal hippocampus were stored in antifreeze (30% glycerol/30% ethylene glycol) at −20 °C until they were processed for immunohistochemistry. For Arc immunohistochemistry, selected tissue sections were washed in 0.1 M PBS and incubated in quenching solution (30% H202), washed and incubated in blocking solution for 1 h at room temperature (5% normal goat serum, 0.4% Triton X-100, and 1% bovine serum albumin). Sections were then incubated overnight at 4 °C in primary antibody (anti-Arc, 1:1000, Synaptic Systems). The next day, sections were washed in 0.1 M PBS and incubated in biotinylated secondary antibody (goat anti-rabbit, 1:200, Invitrogen) for 1 h at room temperature. After three washes, staining was amplified using VectaStain ABC kit (Vector Laboratories) and developed in DAB peroxidase substrate (Vector Laboratories). Sections were mounted on electrostatic slides (Fisher) and coverslipped with permount. Arc cell counts for lateral amygdala (LA) and dorsal dentate gyrus (DG) were manually scored using NIH ImageJ by trained observers blind to experimental conditions. Averages from each region and each hemisphere within subject were averaged as one value, counted from three sections per animal.

### Anatomical tracing and activation analysis

To determine injury-induced changes in activity within LA projecting neurons that are known to be required for auditory fear, we looked at Arc expression in LA projecting neurons during white noise exposure after FPI. Three weeks prior to FPI surgery, rats (n = 20) received bilateral stereotaxic infusions of cholera toxin B (CTB; ThermoFisher; 5 µg/µl) into the lateral amygdala (LA; A/P: −2.7 mm; M/L: + 5.0 mm; D/V: −8.2 mm relative to bregma). Similar to the previous experiments, 48 h after FPI, all rats were exposed to 7 trials of 75 dB white noise and 90 min later rats were perfused. Brains were processed for Arc immunofluorescence histochemistry. Selected tissue sections (40 µm were washed in 0.1 M PBS and incubated in blocking solution for 1 h at room temperature (5% normal goat serum, 0.4% Triton X-100). Sections were then incubated overnight at 4 °C in primary antibody (anti-Arc, 1:1000, Synaptic Systems). The next day, sections were washed in 0.1 M PBS and incubated in secondary antibody (goat anti-rabbit Alexa fluor 594, 1:500, Life Technologies) for 1 h at room temperature. Sections were washed in 0.1 M PBS, mounted on electrostatic slides (Fisher), and coverslipped with Vectashield (Fisher Scientific). Percentage of Arc + cells in LA-projecting cells (CTB + ) was analyzed in regions that provide auditory information to the LA^79^, the auditory thalamus (medial geniculate nucleus; MGN) and secondary auditory cortex (Te3).

### Data analysis

Behavioral data were analyzed using mixed factors analysis of variance (ANOVA) for injury group (sham, FPI) across trials. Arc data were analyzed by a two way between groups multivariate ANOVA for injury group (sham, FPI), and noise condition (no noise, + noise) as independent variables for each brain region and hemisphere separately (LA and dorsal DG for both ipsilateral (ipsi) and contralateral (contra) relative to injury). CTB-Arc data were analyzed as a multivariate ANOVA for injury across brain regions separately (ipsi and contra MGN and Te3). Statistical significance was determined at p-value of 0.05 or less, and when significant interactions were detected, post hoc analyses were performed for simple main effects.

## Supplementary information

Supplemental Materials
