# Supplementary material for: Sensory sensitivity as a link between concussive traumatic brain injury and PTSD
Source: Sci Rep. 2019 Sep 25;9:13841. doi: 10.1038/s41598-019-50312-y (PMC6761112; doi:10.1038/s41598-019-50312-y)
Supplement: Supplementary file 1 — Supplemental Materials [file 41598_2019_50312_MOESM1_ESM.docx]

**Supplemental Material**

*Sensory sensitivity as a link between concussive traumatic brain injury and PTSD*

Ann N. Hoffman, Jamie Lam, David A. Hovda, Christopher C. Giza, &

Michael S. Fanselow

**Figure S1. Similar injury severities across experiments.** Toe pinch withdrawal latency as a measure of loss of consciousness (LOC) after FPI (fluid percussion injury) impact was similar within injury groups across experiments. Mean (269.2s) and median (261.5) LOC for FPI are within the range of mild-moderate level of injury (standard deviation, 142.33). Data are represented as mean ± SEM.


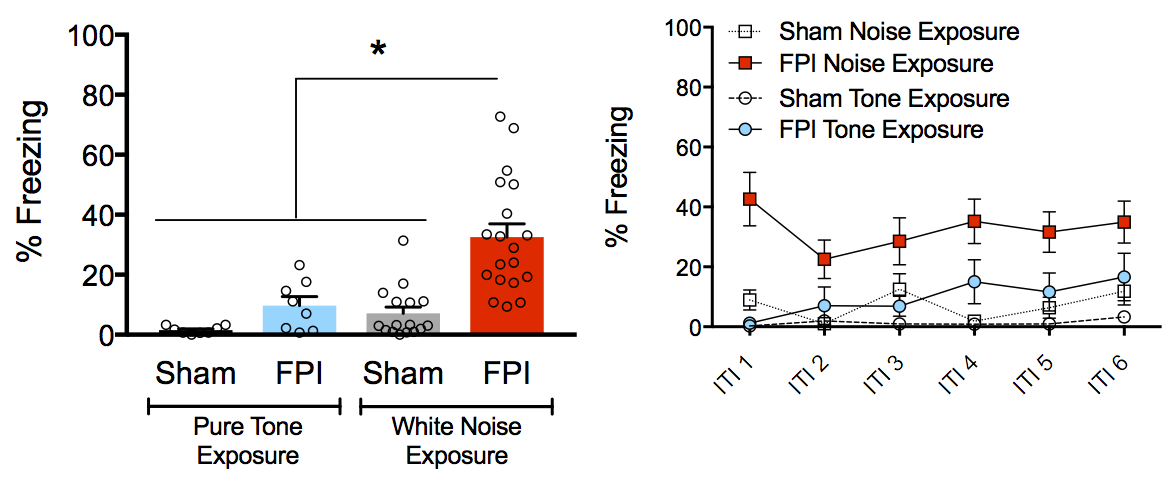


**Figure S2. Stimulus-specific evoked defensive behavior with white noise following FPI.** Comparison of context freezing between auditory stimuli trials by tone vs. white noise exposure following FPI (fluid percussion injury) or sham surgery (session average, left; across trials, right). Although FPI groups displayed statistically elevated levels of freezing between pure tone trials (75dB/2800Hz) relative to respective shams (p<0.05), white noise evoked robust freezing after FPI (p<0.001 vs sham). When analyzed together, a significant injury x stimulus interaction (p<0.05) revealed stimulus specific effects of auditory sensitivity as measured by defensive posturing after FPI.

**FPI increased reactive astrocytes in brain regions associated with contextual and auditory fear**

Astrocytes are the most abundant cell type in the brain and important for functional regulation of neurons and synaptic plasticity ^1^. Increased reactivity within glia including astrocytes is often observed after TBI in both rodent and human brain tissue ^2,3^. We have recently shown increased amygdala GFAP staining at least one week following FPI ^4^, which has been observed up to 51 days after blast-induced TBI ^5^. Astrocyte reactivity after CNS injury plays a role in neuroinflammation ^6^, and there is evidence that neuroinflammation affects behaviorally relevant Arc induction in the hippocampus ^7^. In the same animals as the Arc experiment, we also looked at glilal fibrillary acidic protein (GFAP) expression to determine the level of reactive astrocytes in regions of interest that process auditory and contextual fear in response to white noise after FPI. Immunohistochemistry (IHC) methods were similar to Arc IHC for where frozen sections (40μm) containing the amygdala, dorsal hippocampus, and auditory cortex were stored in antifreeze (30% glycerol/30% ethylene glycol) at -20C until they were processed GFAP IHC. Sections were washed in 0.1M PBS and incubated in quenching solution (30% H202), washed and incubated in blocking solution for 1h at room temperature (5% normal goat serum, 0.4% Triton X-100, and 1% bovine serum albumin). Sections were then incubated overnight at 4ºC in primary antibody (anti-GFAP, 1:10,000, Dako Corporation). The next day, sections were washed in 0.1M PBS and incubated in biotinylated secondary antibody (goat anti-rabbit, 1:200, Invitrogen) for 1h at room temperature. After three washes, staining was amplified using VectaStain ABC kit (Vector Laboratories) and developed in DAB peroxidase substrate (Vector Laboratories). Sections were mounted on electrostatic slides (Fisher), dehydrated, and coverslipped. GFAP staining for the same subregions was qualitatively assessed as percent area stained using ImageJ. Averages from each region and each hemisphere within subject were averaged as one value.

Multivariate analysis across regions and hemispheres for injury and noise condition revealed a significant overall increase in relative GFAP expression in FPI groups, (Wilks’ Lambda test, F(8,10)=6.618, p=0.004). The table below outlines the between groups statistical outcomes for injury effect on GFAP expression for each region, data depicted in figure S1. For regional effects of injury on GFAP, all regions measured revealed bilateral elevations in GFAP expression except for the contralateral medial geniculate, which was not different overall from sham.

| Region | Result | Significance (Sham vs. FPI) |
| --- | --- | --- |
| Lateral amygdala ipsi | F(1, 17)=44.649, p<0.001 | *** |
| Lateral amygdala contra | F(1,17)=15.348, p=0.001 | *** |
| Dentate gyrus ipsi | F(1, 17)=7.6, p=0.013 | * |
| Dentate gyrus contra | F(1,17)=10.442, p=0.005 | ** |
| Auditory Cortex ipsi | F(1,17)=26.611, p<0.001 | *** |
| Auditory Cortex contra | F(1,17)=6.905, p=0.018 | * |
| Medial geniculate ipsi | F(1,17)=22.013, p<0.001 | *** |
| Medial geniculate contra | F(1,17)=0.501, p=0.489 | ns |

No significant effects were observed for noise condition or the interaction, however GFAP in the ipsi LA was nonsignificantly increased in the noise + groups compared to quiet controls (F(1,17)=2.476, p=0.061). It is interesting to speculate whether white noise exposure, especially after injury, affects glial activation in sensory-emotional processing circuitry and whether such an effect would influence neuronal activity. It may be the case that 90min after a single exposure would be too early to detect such an effect. In any case, changes in Arc induction, GFAP expression, and increased defensive behavior in response to white noise after FPI indicate dysregulated cognitive-emotional processing in the presence of this noxious auditory stimulus perceived by the TBI brain.

**
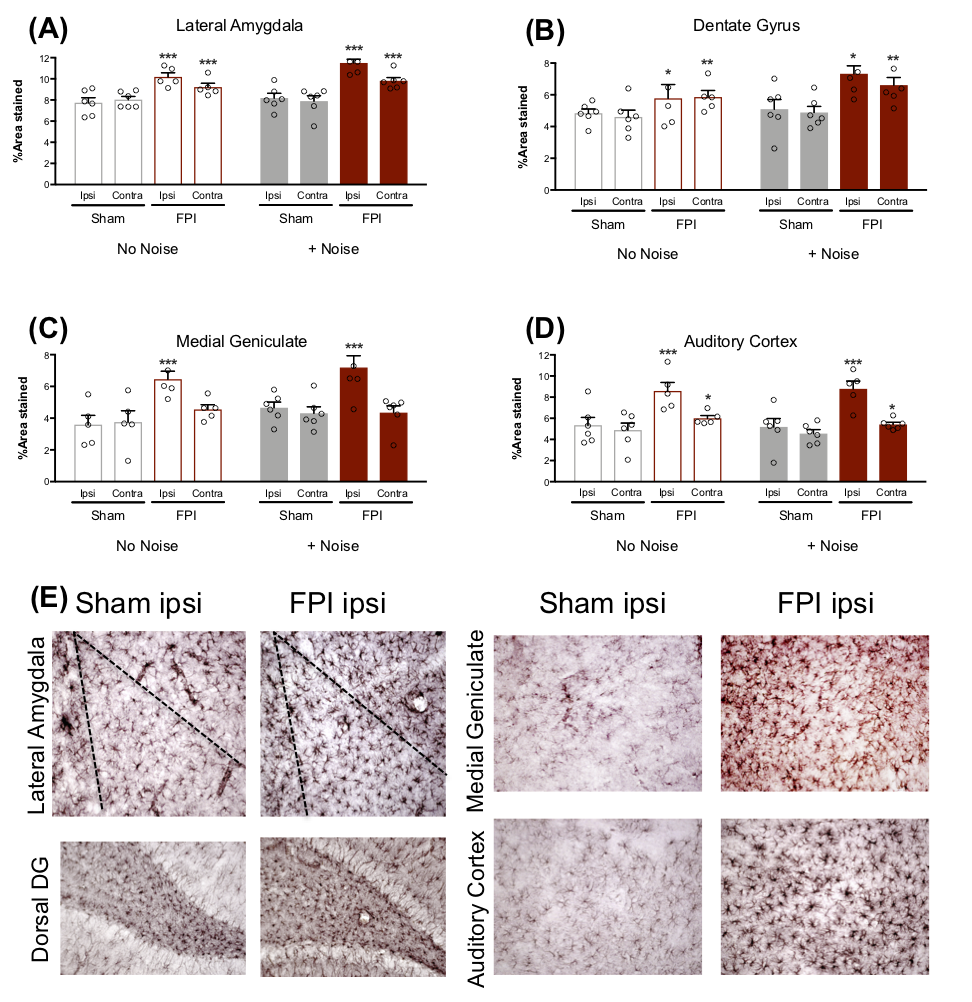
**

**Figure S3. Increased astrocyte reactivity by GFAP expression following FPI**. Increased GFAP expression in bilateral LA (A), dorsal dentate gyrus (B), and auditory cortex (D) was observed across FPI groups compared to sham regardless of white noise exposure. In the medial geniculate nucleus, increased GFAP expression was observed only in the ipsilateral hemisphere (C). Representative images of GFAP staining across ipsilateral (left) regions (4x magnification). Data are represented as mean ± SEM; ***p<0.001; **p<0.01; *p<0.05 vs. Sham.

**Corroboration of two IEGs: Increased activity in ipsilateral thalamo-amygdala projecting neurons during white noise exposure after lateral FPI with c-Fos and Arc**

While the data included in the main manuscript support that Arc induction in the LA is driven by Arc activity in MGN neurons projecting to LA, we further supported this effect with a different and commonly used IEG, c-Fos. A separate series of tissue were from the projection mapping experiment and stained for c-Fos. Neurons projecting to LA positive for CTB expression in MGN and Te3 were co-stained for c-Fos similarly to Arc IHC methods for immunofluorescence. Briefly, for c-Fos immunohistochemistry, selected tissue sections were washed in 0.1M PBS and incubated in blocking solution for 1h at room temperature (5% normal goat serum, 0.4% Triton X-100). Sections were then incubated overnight at 4ºC in primary antibody (anti-c-Fos, 1:10,000, Millipore). The next day, sections were washed in 0.1M PBS and incubated in secondary antibody (goat anti-rabbit Alexa fluor 594, 1:500, Life Technologies) for 1h at room temperature. Sections were washed in 0.1M PBS, mounted on electrostatic slides (Fisher), and coverslipped with Vectashield (Fisher Scientific). Percentage of c-Fos+ cells in LA-projecting cells (CTB+) was analyzed in regions that provide auditory information to the LA ^8^, the auditory thalamus (medial geniculate nucleus; MGN) and secondary auditory cortex (Te3). For c-Fos expression in CTB+ neurons projecting to LA, multivariate analyses (MANOVA) revealed a significant between groups effect of injury in the ipsi MGN, (F(1,8)=7.134, p=0.028), with no other significant effects observed.


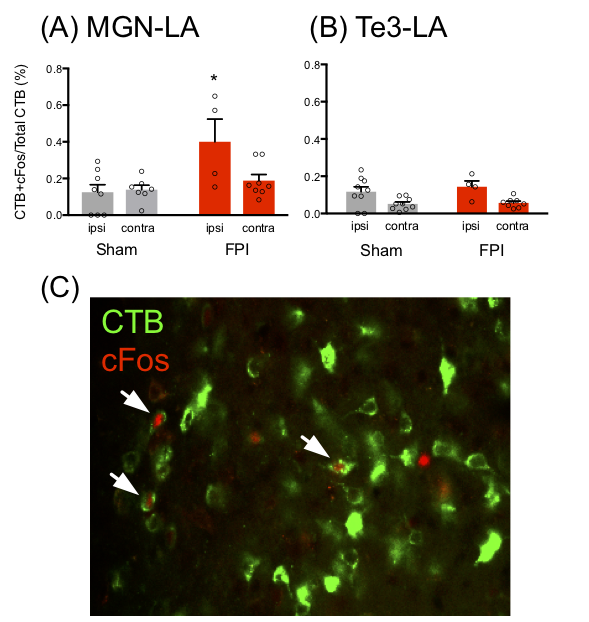
**Figure S4. Increased c-Fos activity in ipsilateral thalamo-amygdala projecting neurons during white noise exposure after lateral FPI.** Increased c-Fos activity in ipsi MGN-LA (A) projecting neurons but not Te3-LA (B) during white noise exposure after FPI. Representative c-Fos activity (red) in retrolabeled CTB in LA afferents (green); 20x magnification. Data are represented as mean ± SEM; *p<0.05 vs. Sham.

References

1 Pekny, M. & Pekna, M. Astrocyte reactivity and reactive astrogliosis: costs and benefits. *Physiological Reviews* **94**, 1077-1098, doi:10.1152/physrev.00041.2013 (2014).

2 Burda, J. E., Bernstein, A. M. & Sofroniew, M. V. Astrocyte roles in traumatic brain injury. *Experimental neurology* **275 Pt 3**, 305-315, doi:10.1016/j.expneurol.2015.03.020 (2016).

3 Hausmann, R., Riess, R., Fieguth, A. & Betz, P. Immunohistochemical investigations on the course of astroglial GFAP expression following human brain injury. *International Journal of Legal Medicine* **113**, 70-75 (2000).

4 Hoffman, A. N. *et al.* Early and Persistent Dendritic Hypertrophy in the Basolateral Amygdala following Experimental Diffuse Traumatic Brain Injury. *Journal of neurotrauma*, doi:10.1089/neu.2015.4339 (2016).

5 Kovesdi, E. *et al.* Acute minocycline treatment mitigates the symptoms of mild blast-induced traumatic brain injury. *Frontiers in neurology* **3**, 111, doi:10.3389/fneur.2012.00111 (2012).

6 Laird, M. D., Vender, J. R. & Dhandapani, K. M. Opposing roles for reactive astrocytes following traumatic brain injury. *Neurosignals* **16**, 154-164, doi:10.1159/000111560 (2008).

7 Rosi, S. Neuroinflammation and the plasticity-related immediate-early gene Arc. *Brain, Behavior, and Immunity* **25 Suppl 1**, S39-49, doi:10.1016/j.bbi.2011.02.003 (2011).

8 Maren, S. & Quirk, G. J. Neuronal signalling of fear memory. *Nat. Rev. Neurosci.* **5**, 844-852, doi:nrn1535 [pii]

10.1038/nrn1535 (2004).
